# Progress of Bacteriophage Research and Application in the Treatment of Bovine Mastitis: A Review

**DOI:** 10.3390/vetsci13080783

**Published:** 2026-08-04

**Authors:** Jingyi Gao, Yuhan Ding, Aoxiang He, Wanyan Zhang, Huaqun Chen

**Affiliations:** School of Life Sciences, Nanjing Normal University, Nanjing 210023, China; 09230440@njnu.edu.cn (J.G.); 09230201@njnu.edu.cn (Y.D.); 09230513@njnu.edu.cn (A.H.); 251212033@njnu.edu.cn (W.Z.)

**Keywords:** bacteriophages (phages), bovine mastitis, pathogens, phage therapy

## Abstract

Bovine mastitis, caused by both contagious and environmental pathogens, represents a major infectious disease burden in the global dairy industry. Antibiotics remain the primary treatment option, but their effectiveness is limited by the blood–milk barrier, drug residues, and the growing threat of multidrug-resistant bacteria. Among the various alternatives, phage therapy has drawn particular attention due to its specificity, ability to disrupt biofilms, low impact on commensal flora, and self-replication at infection sites. In this review, we summarize recent advances in the isolation of lytic phages targeting major mastitis-causing pathogens, their bactericidal mechanisms, and their performance in vitro and in vivo. We also discuss key obstacles to clinical translation including formulation stability, narrow host range, and safety concerns. Moreover, the potential strategies to overcome these issues are explored. This review provides a useful reference for research on phage therapy against bovine mastitis.

## Simple Summary

Bovine mastitis remains a major problem in the dairy industry, and antibiotic use is increasingly limited by resistance and residues. Phage therapy is gaining interest as a potential alternative. This review summarizes recent findings on lytic phages against common mastitis pathogens, their antibacterial mechanisms, and available in vivo results. We also discuss key barriers, including formulation stability and host range, as well as current challenges and potential strategies to overcome these issues.

## 1. Introduction

Dairy farming is a cornerstone of the global livestock industry, providing high-quality dairy products for human consumption while serving as an economic pillar in many countries and regions. However, bovine mastitis, one of the most prevalent and economically devastating diseases in dairy production, continues to impede the sustainable development of the sector [1,2]. Typically caused by microbial infection, mastitis leads to altered milk composition, reduced milk yield, increased culling rates, and elevated treatment costs [3,4]. It ranks among the most costly disease categories in dairy farming, accounting for global annual losses of approximately USD 22 billion, which represents more than one-third of the industry’s total economic losses [5]. More critically, mastitis poses broader concerns regarding milk quality, safety, and public health. The widespread use of antimicrobials in dairy production is accelerating the dissemination of antimicrobial resistance (AMR) across the animal–food–human interface, constituting a latent threat from a One Health perspective.

Antibiotic therapy remains the primary intervention for bovine mastitis, commonly delivered through intramammary infusion or systemic administration to control infection [6]. Nevertheless, the limitations of conventional antibiotic therapy are becoming increasingly evident. On one hand, the unique anatomical and physiological architecture of the mammary gland forms a natural barrier to drug delivery, the blood–milk barrier, which restricts drug distribution within the gland. Moreover, periodic milking results in intermittent drug elimination rather than sustained exposure, and milk constituents can inactivate certain drugs, leading to insufficient drug concentrations at the site of infection and compromised therapeutic efficacy [3]. On the other hand, the escalating problem of antimicrobial resistance continues to narrow treatment options. Key mastitis pathogens, such as *Staphylococcus aureus* (*S. aureus*) and *Streptococcus* species, can further evade antibiotic killing through mechanisms including intracellular persistence and biofilm formation, contributing to persistent infections and high recurrence rates [3,6,7]. Consequently, the exploration of novel anti-infective strategies has become an urgent imperative in the field of bovine mastitis prevention and control.

Phage therapy has recently regained attention as a potential alternative to antibiotics. Bacteriophages are natural viruses that specifically lyse bacteria, offering distinct advantages such as high target specificity, the ability to penetrate biofilms, the absence of drug residues, and minimal disruption to the host’s normal microbiota [2,8]. A growing body of research on the isolation and application of phages targeting the principal mastitis-causing pathogens has yielded encouraging progress, demonstrating considerable promise for practical application [2,6,9].

Based on a systematic review of the major etiological pathogens of bovine mastitis and current major treatments along with their limitations, this review will focus on recent advances in isolation of virulent phages targeting major mastitis-causing pathogens, their bactericidal mechanisms, and their in vitro and in vivo efficacy. We also discuss the challenges confronting phage therapy and potential strategies. Specifically, we aim to assess the translational potential of phage therapy by examining the evidence across phage isolation, bactericidal mechanisms, efficacy studies, and clinical barriers. Both the progress made and the gaps that currently limit clinical application are identified. It is hoped that this review will serve as a reference for subsequent research and translation, and contribute to the precision control of bovine mastitis in the “post-antibiotic era” and the realization of “antibiotic-free dairy” production.

## 2. Types and Epidemiological Distribution of Major Pathogens of Bovine Mastitis

**Figure 1:**
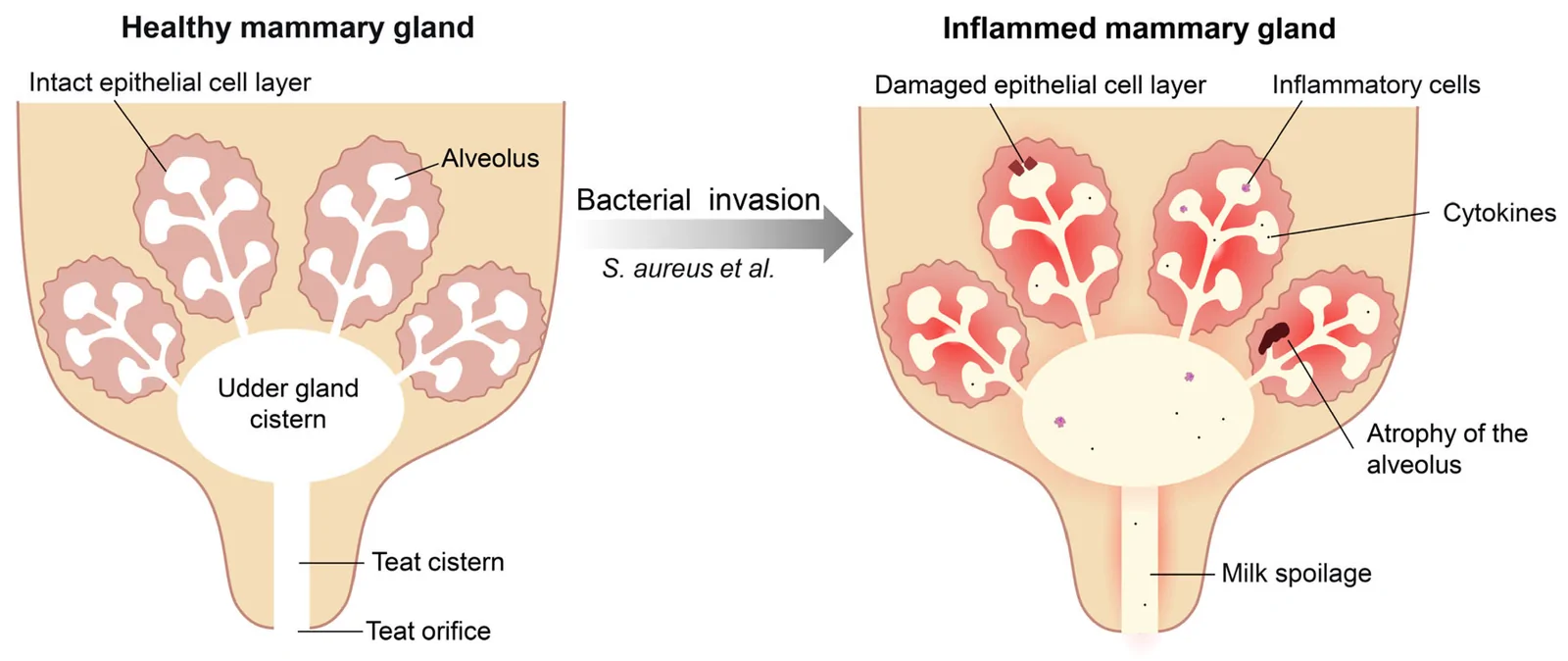
Schematic diagram of a healthy bovine mammary gland and an inflamed mammary gland induced by bacterial invasion. Pathogenic bacteria, including *Staphylococcus aureus*, ascend along the mammary ducts and trigger a local inflammatory response, characterized by immune cell infiltration and pro-inflammatory cytokine secretion, which subsequently leads to epithelial damage, acinar atrophy, and impaired milk quality.

Bovine mastitis is caused by a diverse array of pathogens. Bacterial infection of the bovine mammary gland triggers an inflammatory response, driving the pathological progression of mastitis in cows [10]. The inflammation of the mammary gland is illustrated in Figure 1. Based on their transmission routes and ecological niches, these pathogens fall into two main categories: contagious and environmental [11]. These two categories differ significantly in their epidemiological characteristics and infection patterns.

### 2.1. Contagious Pathogens

Contagious pathogens primarily spread among cows during milking through milking machines, operators’ hands, or towels. They colonize within the mammary gland and cause chronic or subclinical infections that are difficult to eradicate. *Staphylococcus aureus*, *Streptococcus* species and *Mycoplasma bovis* are typical contagious pathogens attributed to bovine mastitis.

*S. aureus* is currently the foremost contagious pathogen inducing subclinical mastitis in dairy cows globally and is also a key pathogenic strain causing chronic, recurrent infections [12,13]. Epidemiological survey data indicate that in farms with poor management, the isolation rate of this pathogen can exceed 30% in China [14]. In recent years, the prevalence of methicillin-resistant *S. aureus* (MRSA), particularly multidrug-resistant MRSA isolates, has continued to increase, posing a significant challenge to conventional antibiotic treatment regimens [15].

*Streptococcus agalactiae* (*S. agalactiae*) is a typical obligate mammary pathogen whose transmission occurs mainly through milking procedures [16]. In contrast, *Streptococcus dysgalactiae* and *Streptococcus uberis* can be transmitted both through milking and environmental routes, such as bedding and feces.

*Mycoplasma bovis* is an important pathogen causing refractory mastitis in dairy cows. In large-scale intensive farming environments, this pathogen can readily trigger mastitis outbreaks. Conventional antibiotic therapy is often ineffective against *Mycoplasma bovis*, directly leading to the culling of affected cows [17]. In some regional surveys, the isolation rate of *Mycoplasma bovis* was reported to be approximately 10–15% [18].

### 2.2. Environmental Pathogens

Environmental pathogens mainly originate from the cows’ living environment, including bedding, manure, and drinking water. Their infection is often related to environmental hygiene management, lactation stage, and seasonal factors. *Escherichia coli* (*E. coli*) and *Klebsiella pneumoniae* (*K. pneumoniae*) are the primary environmental pathogens involved.

*E. coli* infection is associated with acute clinical mastitis in dairy cows, with a high incidence during the peak lactation period and around the dry period [19,20]. Among clinical mastitis cases, the isolation rate of *E. coli* is approximately 25–35%, and the risk of infection may increase during warm and humid periods [21]. Some *E. coli* strains carry multiple virulence genes that can induce severe systemic symptoms in affected cows, leading to death [19].

Recently, *K. pneumoniae* infections have continued to rise in large-scale intensive farming operations, making it the second most important environmental pathogen. Affected cows typically exhibit severe clinical symptoms that are difficult to cure. This is mainly attributed to three factors: first, its generally low sensitivity to antibiotics; second, its thick capsular polysaccharide structure leading to immune escape by blocking complement binding and reducing the phagocytic efficiency of neutrophils and macrophages; third, its high propensity to form biofilms [22].

Currently, *S. aureus* and *S. agalactiae* remain the primary pathogen groups causing bovine mastitis [12,16,23]. Long-term antibiotic abuse has accelerated the emergence and accumulation of drug-resistant strains, further increasing the difficulty of clinical treatment of bovine mastitis [3].

## 3. Advances in the Treatment of Bovine Mastitis

Antibiotics remain the first-line treatment, but their limitations have driven growing interest in non-antibiotic alternatives. This section reviews conventional antibiotics and non-antibiotic strategies, and highlights phage therapy as a particularly promising approach for bovine mastitis.

### 3.1. Antibiotic Therapy

Commonly used antibiotics for bovine mastitis include penicillin, sulfonamides, ampicillin, cloxacillin, and aminoglycosides [24]. These drugs exert antibacterial effects through mechanisms such as blocking bacterial cell wall synthesis, interfering with protein synthesis, or inhibiting nucleic acid synthesis.

Antibiotic therapy has significant limitations in agricultural production applications. First, antibiotics struggle to maintain effective concentrations at the site of mammary gland infection due to milk components and pharmacokinetic factors, particularly in chronic and subclinical cases [3]. Second, biofilm formation physically protects bacteria, severely compromising therapeutic efficacy. Third, drug resistance remains a core challenge, with major mastitis pathogens (e.g., *S. aureus*, coagulase-negative *Staphylococci*, and *S. agalactiae*) being resistant to multiple classes of antibiotics [25,26,27]. In addition, antibiotic residues pose public health risks [28].

### 3.2. Non-Antibiotic Therapies

A variety of non-antibiotic strategies have recently been investigated for the management of bovine mastitis, including probiotics, bacteriocins, vaccines, nanoparticles, and phage therapy. These approaches operate through distinct mechanisms and offer potential benefits such as reducing antibiotic reliance, minimizing drug residues, and improving the safety of dairy products. Probiotics, for example, have long been applied in poultry production and are now attracting increasing interest in the dairy sector. Although lipopolysaccharides derived from gut microbiota have been associated with mastitis through impairment of the udder barrier, short-chain fatty acids appear to exert protective effects [29,30]. Nevertheless, findings from pilot studies on probiotic efficacy remain inconsistent [31,32]. Bacteriocins such as nisin and bovine HC5 demonstrate potent bactericidal activity in vitro; however, their stability within the mammary gland environment is often suboptimal [8,33,34,35,36]. Vaccines targeting virulence factors hold promise as antibiotic alternatives, yet their cross-protective efficacy against diverse strains remains insufficient [12]. Nanoparticles also exhibit potential for antibacterial activity and targeted drug delivery, although concerns regarding cost and safety persist [37,38,39,40]. In summary, each option presents certain advantages, but also significant limitations. The advantages, limitations, and applicable scenarios of the above-mentioned therapeutic strategies are summarized in Table 1.

Among these strategies, phage therapy stands out as a particularly promising approach. By specifically infecting and lysing host bacteria, phages exhibit potent activity against major mastitis-associated pathogens. Their ability to replicate at infection sites, disrupt biofilms, and preserve commensal microbiota offers distinct advantages over broad-spectrum alternatives. As multidrug resistance continues to rise globally, phages are increasingly recognized as a viable alternative to conventional antibiotics. Recent advances in formulation, delivery systems, and field evaluations have further underscored their translational potential, positioning phage therapy as a practical and promising strategy for mastitis management in dairy production.

## 4. Bacteriophage Therapy

Bacteriophages are bacterial viruses that infect, lyse, and kill their hosts. Virulent phages, which lyse host cells upon infection, are the type generally employed for therapy. Phage therapy has been used to treat human infections in countries such as Georgia and Russia and is advancing toward regulatory approval in Europe and China [52,53]. In veterinary medicine, phage therapy for bovine mastitis has been under investigation for many years, with numerous phages targeting mastitis-causing pathogens reported to date. The vast majority of these phages belong to the order *Caudoviricetes*, likely due to their abundance in nature and their tail structures that enable efficient recognition and lysis of major mastitis pathogens [54]. This section covers phage isolation and screening, bactericidal mechanisms, efficacy and safety evaluation, pharmacokinetics, and the challenges facing clinical translation.

### 4.1. Sources of Bacterial Strains for Bovine Mastitis Phage Screening

Phage screening starts with the selection of host bacterial strains. The source of these strains determines the clinical relevance of the phages obtained. Bacterial strains from bovine mastitis cases used for screening phages against specific bacteria are typically natural strains isolated and identified directly from clinical or environmental samples [55]. This is also the most commonly used source of bacterial strains for bovine mastitis phage screening. In addition, some research groups conduct studies using standard strains purchased from culture collections [56].

### 4.2. Isolation, Screening, and Efficacy of Bovine Mastitis Phages

The isolation of lytic phages against mastitis pathogens follows a standard plaque assay workflow, but the criteria for selecting candidate phages vary considerably across studies. This subsection summarizes current isolation practices, the functional parameters typically evaluated, and the distribution of research efforts across different mastitis pathogens.

Phages screened against bovine mastitis pathogens are typically virulent phages, and the plaque assay involves mixing the host bacteria with the test sample, embedding the mixture in semi-solid agar, and observing plaque formation [9,55].

The Lytic bovine mastitis phages are primarily screened from environmental samples using plaque assays, with dairy farm wastewater, milk, feces, and raw milk serving as the main sources [57,58]. Regardless of source, candidate phages for therapeutic use must meet a set of inclusion criteria to be considered suitable for further development. These typically include broad host range (or at least activity against epidemiologically relevant strains), strong lytic activity, good environmental stability (e.g., pH and thermal tolerance), absence of virulence and lysogeny-related genes in the genome, and safety in both in vitro and in vivo anti-infective studies.

To facilitate the systematic evaluation of candidate phages, standardized genomic screening workflows have been established. The Sphae pipeline, for example, provides an automated approach for detecting virulence factors, antimicrobial resistance genes, integrases, recombinases, and other lysogeny-associated markers that could preclude therapeutic use [59]. Strict exclusion of temperate phages is widely adopted as a primary criterion, as their ability to integrate into the host chromosome and mediate horizontal gene transfer via generalized or specialized transduction poses a well-documented safety risk. The ISO standard for phage quality control further mandates genetic sequencing to confirm the absence of toxin and resistance genes [60,61]. While these criteria are widely recognized, the relative weighting assigned to each parameter varies considerably across studies, and there is currently no standardized scoring system to prioritize candidates, making cross-study comparison difficult.

Among the major mastitis pathogens, *S. aureus* has received the most attention, and several highly efficient lytic phages have been reported, such as OPT-SA02, OPT-SC01 and OPT-SX11 [62,63]. Phages targeting *E. coli* are the second most extensively studied group, and some have shown significant potential for controlling clinical mastitis. In contrast, phages targeting *K. pneumoniae* and *Streptococcus* species remain relatively underexplored, despite the clinical importance of these pathogens. The current translational status, research characteristics, and supporting references for phages targeting these major mastitis pathogens are summarized in Table 2. This uneven distribution reflects not only differences in research priorities but also the inherent biological differences among bacterial species, including variations in cell wall structure and receptor accessibility, which affect isolation success rates.

From a structural perspective, the vast majority of isolated phages belong to the class *Caudoviricetes* (formerly classified as *Myoviridae*, *Siphoviridae*, and *Podoviridae* under the traditional morphology-based taxonomy). Their tail structures carry receptor-binding proteins (RBPs) that determine host specificity. Long tail fibers (LTFs) mediate reversible recognition of specific surface receptors, while short tail fibers (STFs) are responsible for irreversible attachment to the lipopolysaccharide core. The variability of the LTF tip region accounts for strain-level specificity, whereas STFs are relatively conserved across phages targeting the same species [64]. This structural architecture explains why phages are generally highly specific to particular bacterial strains—a therapeutic advantage in terms of sparing commensal microbiota, but also a limitation when dealing with genetically diverse field isolates.

**Table 2:** Comparison of existing bovine mastitis phages.

Target Pathogen	Current Translational Status	Research Characteristics	References
*E. coli*	dairy cow studies	focus on bactericidal efficacy and therapeutic outcomes; variable responses among phages	[56,65]
*S. aureus* (including MRSA)	field trial	emphasis on MDR strains and biofilm-associated infections; limited clinical validation	[48,55,66]
*K. pneumoniae*	animal models	focus on MDR isolates; limited dairy cow evidence	[67,68]
*Streptococcus* spp.	mainly endolysin-based studies	predominantly in vitro evaluation; limited in vivo validation	[69,70]

Functionally, the isolated phages generally exhibit short latency periods, large burst sizes, good stability in milk, and the ability to penetrate biofilms. Some phages also show potent activity against multidrug-resistant strains, including MRSA. However, the functional characterization across studies remains inconsistent in terms of methodology and reporting standards—for example, burst size and latency are measured under different conditions (temperature, medium, MOI), which limits the comparability of reported values and hinders the selection of lead candidates for further development.

### 4.3. Bactericidal Mechanism of Phages

**Figure 2:**
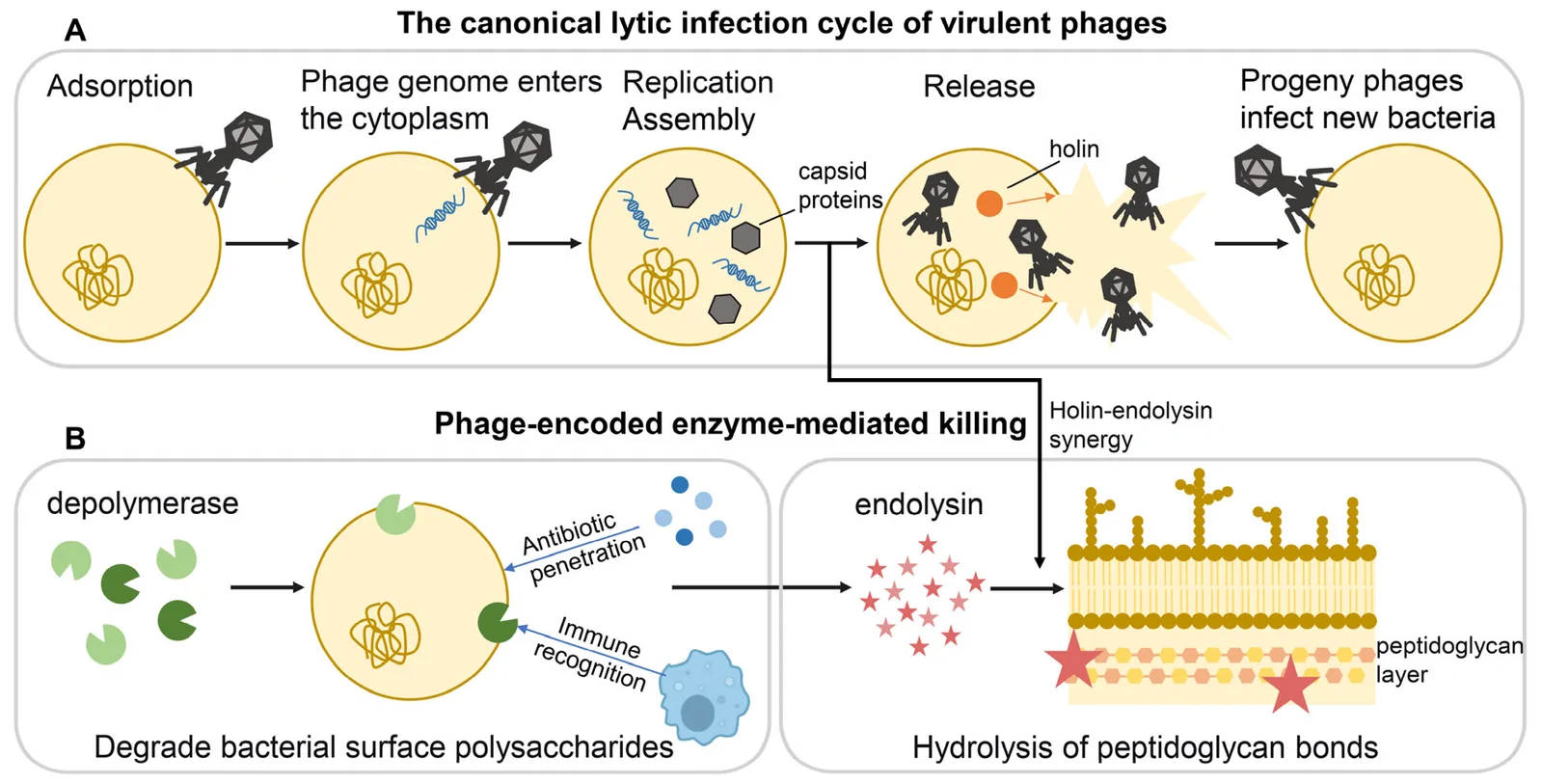
Bactericidal mechanisms of phages and phage-derived enzymes. (**A**). The canonical lytic infection cycle of virulent phages. Phages specifically recognize and adsorb to bacterial surface receptors via tail fiber proteins, followed by the injection of the phage genome into the host cytoplasm. Subsequently, phages hijack the host transcriptional and translational machinery to replicate the viral genome and synthesize structural proteins for the assembly of progeny virions. Ultimately, the coordinated action of holins and endolysins disrupts bacterial membrane integrity and degrades the peptidoglycan layer, resulting in bacterial lysis and the release of progeny phages for secondary infection. (**B**). Phage-encoded functional enzyme-mediated bactericidal effects. Depolymerases degrade the extracellular polysaccharide barriers of bacteria, thereby improving antibiotic penetration efficiency and facilitating host immune recognition and clearance of invasive bacteria. Exogenously added endolysins hydrolyze critical chemical bonds in peptidoglycan, triggering rapid osmotic lysis [68,69,71,72,73,74].

Phages kill bacteria through two routes: the lytic infection cycle and phage-encoded enzymes. The key mechanisms are illustrated in Figure 2 and described in detail below.

The bactericidal action of virulent phages is typically accomplished through a highly ordered process encompassing adsorption, injection, biosynthesis, assembly and release of progeny phages. Phages use their tail fiber proteins to specifically recognize and adsorb to bacterial surface receptors. They then inject their DNA or RNA into the host cell, hijack the host transcription and translation machinery, synthesize middle and late proteins, and assemble progeny phage particles. Ultimately, bacterial lysis is achieved through the synergistic action of holins, which form non-specific pores in the cell membrane, and endolysins (also termed lysins), which degrade the peptidoglycan of the cell wall [70,71]. Some phages can also initially enter a lysogenic cycle, integrating their genome into the bacterial chromosome, and switch to the lytic cycle upon induction by specific environmental signals.

Beyond intact phages, individual functional proteins encoded by phages themselves also possess considerable bactericidal potential. Endolysins, as phage-derived peptidoglycan hydrolases, are capable of rapidly killing bacteria both in vitro and in animal models [72]. These enzymes hydrolyze key chemical bonds in the bacterial cell wall peptidoglycan, such as β-1,4-glycosidic bonds or amide bonds, leading to bacterial lysis driven by osmotic pressure. The advantages of endolysins include rapid action, high specificity, and a low propensity for resistance development. Depolymerases are enzymes that target the polysaccharide barrier on the bacterial surface. Their core value lies in degrading this polysaccharide physical barrier, thereby enhancing the penetration efficacy of antibiotics or endolysins, and promoting the recognition and clearance of bacteria by the host immune system [73].

### 4.4. Evaluation of Phage Efficacy and Safety

Candidate phages must be validated at multiple levels, from in vitro assays to animal models and field trials. This subsection reviews the experimental systems used to assess efficacy and safety, and summarizes the key findings from each.

For in vitro antibacterial efficacy assessment, researchers usually employ standardized model systems to systematically determine key phage parameters, including multiplicity of infection (MOI), latent period, burst size, and environmental stability [55,56].

For in vivo efficacy validation, the mouse mastitis model has become one of the primary tools for the preclinical evaluation of phage therapy. This model typically uses lactating specific-pathogen-free (SPF) female mice. Under anesthesia, infection is established by injecting target pathogens in the logarithmic growth phase through the abdominal mammary duct. At 12 h post-infection, phage, antibiotics, or PBS are administered via the same route. Mice are euthanized 72 h after treatment, and mammary gland tissues are collected for bacterial load quantification, histopathological evaluation, detection of inflammatory cytokine expression, analysis of blood–milk barrier integrity, and assessment of phage biodistribution and metabolism [67]. Mouse model studies have demonstrated that phage administration significantly reduces the pathogen load in mammary tissue, markedly decreases the expression levels of pro-inflammatory cytokines such as TNF-α, IL-1β and IL-6, and notably alleviates histopathological damage [63,75]. These animal model studies provide important evidence for the efficacy and safety of phage therapy. Equally critical is efficacy evaluation under real-world production conditions. Recently, the group of Krömker conducted a randomized, negative-controlled field trial in dairy cows infected with *S. aureus* to directly assess the safety and efficacy of phage cocktail therapy. The results revealed a bacteriological cure rate of 81.3% in the treatment group, compared with a spontaneous cure rate of only 28.6% in the untreated control group. This study not only confirmed the good tolerability and safety of phage therapy under practical production conditions but also provided key evidence for the clinical application of phage preparations in the treatment of bovine mastitis [66].

A number of bacteriophage products have already received regulatory approval or reached the market in various countries. Table 3 summarizes representative examples, including commercially available phage products as well as one candidate formulation for bovine mastitis that is currently undergoing field trials. These cases point to the translational feasibility of phage-based approaches and may inform future developments in mastitis therapy.

### 4.5. Current Phage-Based Therapeutic Approaches for Bovine Mastitis

Several phage-based strategies have been explored for bovine mastitis treatment, each with distinct advantages and limitations.

Single-phage therapy employs one specific phage to infect and lyse the target pathogen [9]. The advantage of this approach lies in its well-defined composition. In terms of efficacy, single phages display potent lytic activity initially against target strains [55,62].

Phage cocktail therapy, which combines multiple phages, has become a major research focus. Guo et al. provided key in vivo evidence for this strategy using a cocktail composed of three *E. coli* phages to treat mastitis caused by drug-resistant *E. coli* in dairy cows. Compared with the untreated group, the cocktail-treated group exhibited significantly reduced bacterial loads and somatic cell counts (SCC), along with alleviated clinical signs, achieving efficacy comparable to that of the antibiotic group [56,66]. Królikowska et al. proposed a novel cocktail design strategy based on in-depth bioinformatics analysis and in vitro screening, targeting both *E. coli* and *S. aureus*. They selected five anti-*E. coli* phages and three anti-*S. aureus* phages to formulate a cocktail. In a milk environment model, this cocktail reduced *S. aureus* counts by 45% and *E. coli* counts by 30%, achieved a 99% inhibition rate against *S. aureus* biofilms and over 50% inhibition against biofilms of certain *E. coli* strains [86]. Banar et al. developed a novel broad-spectrum phage cocktail targeting methicillin-resistant *S. aureus* (MRSA), which effectively cleared MRSA infection in a mouse mastitis model, significantly reducing bacterial burden and inflammatory responses [87].

Endolysins are phage-encoded enzymes that lyse the bacterial cell wall from within at the end of the phage infection cycle. Vander Elst et al. characterized two phage-derived endolysins, PlySs2 and PlySs9, which exhibited highly efficient lytic activity in vitro against clinical isolates of *Streptococcus uberis* from bovine mastitis and retained activity in a milk environment [88]. Subsequent work by Schmelcher et al. found that two endolysins derived from streptococcal phages, λSA2 and B30, could also efficiently kill *S. agalactiae* in milk. Moreover, in a mouse mastitis model, the use of an endolysin cocktail demonstrated potent bactericidal effects [69].

Beyond these established strategies, recent advances have addressed intracellular persistence of *S. aureus* and certain *Streptococcus* species in mammary epithelial cells. While phages can be internalized via non-specific pathways, natural uptake is inefficient and leads to lysosomal degradation [89]. To overcome this, liposome encapsulation enables intracellular delivery without phage genetic modification, whereas cell-penetrating peptide engineering modifies capsids (e.g., via CRISPR-Cas9) to enhance internalization [90,91]. These strategies target intracellular reservoirs driving chronic infection and recurrence.

Notably, despite the promising results above, the evidentiary hierarchy warrants distinction. Most evidence for single-phage therapy and endolysins derives from in vitro and murine models [9,88], which cannot fully replicate the bovine udder environment. For phage cocktails, while some in vivo data exist, most evidence similarly relies on rodent models or ex vivo milk models [55,66]. A critical review emphasized that most supporting research has been conducted in model systems rather than in dairy cattle under field conditions [9]. Robust field evidence from randomized controlled trials remains scarce [66]. Therefore, whether these laboratory findings can be translated into effective on-farm applications remains an open question that merits dedicated investigation.

### 4.6. Pharmacokinetics and Pharmacodynamics Studies of Phage Therapy in the Bovine Mammary Gland

The following summarizes available PK/PD data for phages in the bovine mammary gland, along with the key barriers identified to date.

Bacteriophages, as self-replicating antibacterial agents, exhibit pharmacokinetic and pharmacodynamic (PK/PD) properties that differ substantially from those of conventional antibiotics. Their therapeutic effect is considered to depend not only on the administered dose but also on bacterial density and the local microenvironment at the infection site, and this infection-dependent amplification is widely regarded as a key feature of phage PK/PD behavior [92,93].

Studies specifically examining phage PK/PD in the bovine mammary gland remain scarce. The most comprehensive data were obtained from a placebo-controlled trial in which bacteriophage K was administered intramammarily to cows with subclinical *Staphylococcus aureus* mastitis [94]. Following a single dose of 1.25 × 10^11^ PFU per quarter, viable phage remained detectable in milk for up to 36 h post-infusion. However, recovered titers were found to be substantially lower than expected from simple dilution, indicating significant degradation or inactivation of phages within the gland. The bacteriological cure rate was only 16.7%, which was not significantly different from that observed in the saline control group. Additionally, phage infusion into healthy quarters was found to elicit a marked increase in somatic cell count, suggesting local immune activation, whereas this response was not observed in already infected quarters [95]. A subsequent mechanistic study demonstrated that bovine whey proteins inhibit phage adsorption to *S. aureus*, offering a partial explanation for the observed loss of activity in milk [96].

More recent reviews have emphasized that intramammary phage therapy is constrained by PK/PD barriers, including poor tissue penetration, episodic drug elimination via milk flow, and inactivation by milk components, frequently resulting in subtherapeutic exposure at the infection site [3]. These limitations have been noted to be amplified in chronic and subclinical mastitis, where biofilms and intracellular reservoirs further reduce therapeutic efficacy. It has been highlighted that most supporting research has been conducted in model systems rather than in dairy cattle under field conditions, emphasizing the need for bovine-specific PK/PD studies [9]. Collectively, current evidence indicates that while phages can persist in the bovine mammary gland for up to 36 h, their effective concentration is substantially reduced by inactivation and dilution, and therapeutic efficacy in naturally occurring mastitis remains to be definitively demonstrated.

In summary, bovine mammary phage PK/PD data remain scarce. Phages persist in milk for up to 36 h, but effective concentrations are reduced by inactivation and dilution. Cure rates did not differ from controls. Bovine-specific studies are urgently needed.

### 4.7. Challenges and Future Perspectives for Phage Therapy of Bovine Mastitis

Despite considerable progress in vitro and in animal models, clinical translation of phage therapy for bovine mastitis faces multiple hurdles. These challenges and the countermeasures proposed to address them are discussed below.

#### 4.7.1. Host Range Limitation and Phage Resistance

The narrow host range of phages and the rapid emergence of bacterial resistance are two primary problems for mastitis phage therapy. Addressing them requires strategies such as phage cocktails, engineering approaches, and combination with antibiotics. Bovine mastitis is frequently caused by multiple pathogenic species [2,3], and even phages with a broad lytic spectrum may fail to achieve sustained clearance, as bacterial counts often rebound through resistance development. These issues also hinder the standardization and large-scale use of phage-based products [58].

*Host range limitation.* The intrinsic narrow host range of bacteriophages poses a fundamental challenge for mastitis therapy, as most phages infect only a limited spectrum of strains within a single species, determined by receptor-binding proteins (RBPs) that recognize specific surface structures such as lipopolysaccharides, outer membrane proteins, or capsular polysaccharides [2]. Given that bovine mastitis is polymicrobial and caused by diverse pathogens including *Staphylococcus aureus*, *Streptococcus uberis*, *Escherichia coli*, and *Klebsiella* spp. [97], a single phage is insufficient to cover the complex pathogen communities on dairy farms. To address this limitation, phage cocktails combining multiple phages with distinct receptor targets are the most effective approach, as they broaden the lytic spectrum and reduce resistance emergence [9]. Additionally, synthetic engineering of RBPs enables phages to be retargeted to refractory strains, and depolymerases can degrade capsular polysaccharides to expose masked receptors for encapsulated pathogens [64].

*Phage resistance.* Bacteria have evolved diverse defense mechanisms that operate at every stage of the phage infection cycle [98]. At the surface level, bacteria prevent adsorption through receptor modification, including mutations in genes encoding lipopolysaccharides, outer membrane proteins (e.g., OmpC), or capsular polysaccharides (e.g., WcaJ) [58,68]. Intracellular defenses include the restriction-modification (R-M) system that degrades foreign DNA, abortive infection (Abi) mechanisms that trigger infected cell death, and the CRISPR-Cas system that provides adaptive immunity through sequence-specific cleavage [68,99]. The presence of active CRISPR-Cas loci in mastitis-associated *E. coli* isolates has been documented, indicating this endogenous barrier should be assessed when selecting therapeutic phages. Importantly, resistance acquisition incurs fitness costs—reduced growth, attenuated virulence, or restored antibiotic susceptibility [68,98]. This fitness burden creates a therapeutic window for phage-antibiotic synergy (PAS), wherein subinhibitory antibiotics suppress resistant mutants and exploit fitness deficits to resensitize bacteria to conventional agents [99,100]. In mastitis, where intramammary pharmacokinetics are compromised by poor tissue penetration and milk flushing, phage-antibiotic integration offers a complementary strategy to enhance therapeutic durability [3].

In summary, addressing these difficulties requires an integrated approach combining phage cocktails, engineering strategies, and PAS. Predictive modeling of phage–bacteria co-evolution and high-throughput screening may further inform rational design [101]. Future progress will depend on formulation optimization and in vivo validation in cattle models.

#### 4.7.2. Microenvironmental Constraints in the Mammary Gland

The mammary gland environment presents a unique set of biophysical and biochemical barriers to phage therapy. Raw milk contains fat globules, casein micelles, immunoglobulins, and other bioactive proteins, which can interfere with phage adsorption, stability, and lytic activity. In particular, milk fat globules and proteins associated with bacterial agglutination have been reported to inhibit phage–host interactions [58]. Although heat treatment can reduce this inhibitory effect, it is not applicable for in vivo administration [102].

In addition, the inflammatory microenvironment during mastitis poses further challenges. Bovine IgG in mastitic milk promotes *S. aureus* aggregation, reducing phage adsorption by approximately eight-fold and delaying lysis [103,104]. Inflammatory exudates such as proteases and reactive oxygen species may damage phage capsids, while pH alkalinization may affect phage stability. In chronic cases, fibrosis may restrict phage diffusion to deep bacterial reservoirs. Phage infusion into healthy quarters has been reported to increase somatic cell count, suggesting local immune activation [9,95]; whether this leads to phage clearance requires further study. Together, these factors add considerable complexity to phage therapy in the mastitic udder.

To address these constraints, efforts have focused on optimizing phage delivery systems. Encapsulation technologies, including lipid-based nanocarriers and polymeric microparticles, have been explored in animal models to improve phage stability and achieve sustained release at infection sites [105,106]. Phage engineering for enhanced stability in milk is also under investigation. However, pharmacokinetics/pharmacodynamics data in the bovine mammary gland remain scarce. Gill et al. infused phage into healthy cow quarters and observed increased somatic cell count with viable phage still detectable at 36 h. Recovered phage titers were lower than expected from dilution, suggesting inactivation within the gland [95]. These findings highlight the need for further studies on formulation and dosing. The interplay between dosing frequency, milk composition, and phage activity also deserves attention.

#### 4.7.3. Safety and Long-Term Ecological Risks

Beyond efficacy, the safety profile of phage therapy remains a central concern. Currently, safety assessment relies heavily on genomic screening to exclude known virulence determinants and lysogeny-associated genes [60]. While this approach can flag obvious hazards, it does not guarantee that phages will remain benign during in vivo application.

Phages can acquire virulence or antimicrobial resistance genes through horizontal gene transfer (HGT), posing a risk of cross-host transmission, particularly in agricultural settings where livestock, environment, and humans are interconnected [107,108]. To mitigate these risks, standardized genomic screening workflows have been established. The Sphae pipeline, for example, detects virulence factors, resistance genes, integrases, and other lysogeny-associated markers [59]. Strict exclusion of temperate phages is a primary criterion, as their integration into the host chromosome and ability to mediate transduction pose a well-documented HGT risk. ISO standards further mandate genetic sequencing to exclude toxin and resistance genes [60,61].

Perhaps less immediately apparent, but equally consequential, are the long-term ecological risks. Phages are not merely passive killers of target bacteria; they actively shape microbial community dynamics. By imposing strong selective pressure, they may accelerate bacterial adaptive evolution [109], potentially drive the emergence of resistant strains or facilitate the spread of resistance genes within the resident microbiota. This phage-mediated dissemination, if it occurs in the bovine mammary gland or gut, could have lasting effects on the microbial ecology of the animal and its surrounding environment.

What remains most uncertain, however, is the long-term impact of phage therapy on the dairy cows themselves. The current evidence base offers little insight into whether repeated or prolonged phage administration affects host metabolism, disrupts the natural balance of commensal microbiota, or provokes unintended immune responses. Whether they affect the metabolic status of cows, disrupt the homeostasis of the commensal microbiota, or elicit adverse immune responses remains unsupported by large-scale, long-term clinical data [110].

To address these safety concerns, standardized genomic screening workflows (e.g., Sphae) are available to detect virulence factors, resistance genes, and lysogeny markers [59]; strict preference for lytic over temperate phages is widely adopted, with ISO standards mandating genetic sequencing to exclude toxin and resistance genes [60,111]. However, current long-term in vivo safety evaluation systems remain underdeveloped, and larger-scale, extended studies in target species are urgently needed to establish comprehensive safety profiles and guide regulatory approval. The challenges and future perspectives for phage therapy of bovine mastitis are summarized in Table 4.

### 4.8. Regulatory and Commercialization Hurdles

Beyond the scientific challenges, regulatory and manufacturing hurdles also impede clinical translation. The following reviews these barriers and the path forward.

Despite encouraging efficacy results, translating phage therapy for bovine mastitis from laboratory to clinical application faces substantial regulatory and commercialization hurdles. At present, no dedicated regulatory framework exists specifically for phage-based therapeutics. Fragmented approval pathways, complex manufacturing requirements, and stringent quality control standards collectively limit the industrial-scale adoption of phage products in veterinary medicine [52,112]. Moreover, the lack of large-scale, long-term clinical safety evidence has led most countries to permit their use only as placebos or under expanded-access programs [53,113].

*Regulatory Pathways.* The regulatory landscape for veterinary phage products remains fragmented across jurisdictions. In the EU, phage therapies fall under Regulation (EU) 2019/6 and may be classified as veterinary medicinal products, magistral preparations, or food additives, each with distinct requirements not originally designed for phages [52,112]. In the US, therapeutic applications are regulated by the FDA while food safety uses fall under the USDA, with several phages approved via the GRAS pathway [112]. Belgium has adopted a flexible framework allowing hospital exemptions for magistral preparations [52], whereas China remains exploratory, though the 2024 Expert Consensus on Phage Quality Standards represents a first step toward harmonization [114]. The IABS (International Alliance for Biological Standardization) workshop concluded that regulatory harmonization is urgently needed to facilitate market access and reduce development costs [52].

*Manufacturing and Quality Control.* Manufacturing phage products at scale presents unique challenges due to their biological nature [52,112]. Upstream processing requires careful control of MOI, medium composition, temperature and harvest timing. Conditions that work in small flasks often fail in large bioreactors. High-throughput screening and shake-flask trials early in development can help guide scalable process design [115]. Downstream purification presents its own challenges. Removing endotoxins and cellular debris is a major hurdle. Generally used CsCl centrifugation does not scale easily and raises safety concerns. Alternatives such as anion-exchange chromatography with enzymatic treatment have been explored. New methods combining microfiltration, ultrafiltration, and composite chromatography show improved results [116,117]. Phage production also depends on live bacterial hosts which introduces variability. Batch-to-batch inconsistency and storage instability [118,119] remain common problems. Weak patent protection, cheap antibiotics, uncertain returns and limited policy support further reduce commercial interest [120]. On the quality control side, routine checks cover potency (≥10^9^ PFU/mL), purity, genetic screening to exclude harmful genes, and stability under defined storage conditions [114]. Yet for veterinary products, the cost of meeting these standards is often excessively expensive [52,112]. When regulatory oversight is also fragmented, these technical and economic barriers together make it difficult to bring phage therapies into practical use. Phage therapy has real promise but significant hurdles remain—scaling up production, reducing purification costs, ensuring batch-to-batch consistency, improving shelf-life and establishing clearer regulatory pathways. Progress will depend on coordinated efforts from process engineers, regulatory specialists and clinical researchers.

## 5. Conclusions

Bovine mastitis remains a major economic burden on the global dairy industry. Conventional antibiotics are progressively losing efficacy due to the blood–milk barrier, drug residues and rising antimicrobial resistance. Phage therapy has therefore attracted growing interest as a potential alternative. Over the past decades, in vitro studies, rodent models and preliminary field trials have provided encouraging evidence. These studies demonstrate that phages and endolysins can effectively lyse major mastitis pathogens, penetrate biofilms, and show good tolerability in naturally infected cows [66,69]. Advances in synthetic biology and gene editing have further opened possibilities for host range expansion, lytic activity optimization and the integration of diagnostic functions [64,90].

Nevertheless, several limitations must be noted. The current evidence base remains predominantly preclinical. Most data come from in vitro studies, murine models or ex vivo milk experiments [3,9]. Clinical data from naturally infected dairy cows are scarce, and large-scale field trials are lacking. Moreover, challenges such as narrow host range, rapid emergence of phage resistance, regulatory gaps and high manufacturing costs continue to hinder clinical translation [52,64,98,112]. It is therefore more realistic to view phage therapy for now as a complement to antibiotics rather than a replacement.

Prospectively, progress will depend on well-designed field trials in commercial herds to generate robust efficacy data. Research should also prioritize phage cocktail development, host range engineering, improved formulation and delivery systems, and cost-effective production methods. Long-term safety, particularly regarding effects on commensal microbiota, metabolic status and horizontal gene transfer, requires thorough investigation through longitudinal monitoring. Industrial translation will need regulatory-compliant manufacturing and decision-support tools integrated with pathogen surveillance. Realizing the goal of antibiotic-free dairy production will ultimately require coordinated efforts across academia, veterinary practice, industry and regulatory agencies—and this goal, while worth pursuing, remains a long-term aspiration rather than an imminent reality.

## Figures and Tables

**Table 1:** Comparison of non-antibiotic therapeutic strategies for bovine mastitis.

Strategy	Advantages	Limitations	Applicable Clinical Scenarios	References
Probiotic	Competitive exclusion; immune modulation; microecology stabilization; no residue	Strain-specific efficacy; infusion-induced inflammation; low cure rate for chronic infections	Prevention of subclinical mastitis; dry period intervention	[32,41,42]
Bacteriocin	Rapid membrane disruption; low cytotoxicity; no residue	Narrow spectrum (Gram- positive only); high production cost; rapid resistance emergence	Teat dip prophylaxis; adjunctive therapy for mild infections	[8,33,35,36,43]
Vaccines	Induction of specific or cross-protective immune responses; herd-level prophylaxis	Antigenic diversity; immune evasion; weak mammary immunity; no reliable commercial vaccine	Herd-level prophylaxis; reduction in clinical severity	[12,44,45,46]
Nanoparticle	Direct antimicrobial activity; biofilm penetration; drug delivery	High cost; uncertain long-term safety; antibiotic-dependent as carrier	Chronic/biofilm-associated infections; antibiotic potentiation	[37,38,39,40,47]
Phage	Host-specific lysis; anti-MDR efficacy; biofilm disruption; self-amplification; no residue	Narrow host range; milk component interference; temperate phage risk; no regulatory framework	Multidrug-resistant infections; chronic/biofilm-associated infections; antibiotic treatment failure	[9,48,49,50,51]

**Table 3:** Representative bacteriophage products approved or commercially available.

Product	Targets	Primary Application	Product Characteristics	Regulatory Status	References
Sextaphage^®^ (Microgen)	*S. aureus*, *Streptococcus* spp., *E. coli*, *Klebsiella* spp., *Pseudomonas* spp.	Human infectious diseases	Multivalent lytic phage cocktail	ЛП-№(003826)-(РГ-RU)	[76]
IntestiPhage^®^ (Microgen)	*E. coli*, *Shigella* spp., *Salmonella* spp.	Gastrointestinal infections	Oral polyvalent phage cocktail	ЛП-№(004238)-(РГ-RU)	[77]
Pyobacteriophage^®^ (Microgen)	*S. aureus*, *Streptococcus* spp., *E. coli*, *Pseudomonas* spp., *Proteus* spp.	Human wound, respiratory and intestinal infections	Broad-spectrum therapeutic phage cocktail	ЛП-№(006274)-(РГ-RU)	[78]
PhageGuard Listex™ (Micreos Food Safety)	*L. monocytogenes*	Food safety	*Listeria*-specific lytic phage preparation	FDA GRAS (GRN 198/218)	[79]
ListShield™ (Intralytix)	*L. monocytogenes*	Food safety	Lytic phage preparation for food and processing environments	FDA GRAS (GRN 528)	[80]
EcoShield™ (Intralytix)	STEC, incl. *E. coli* O157:H7	Food safety	Multi-phage cocktail targeting pathogenic *E. coli*	FDA GRAS (GRN 834)	[81]
SalmoFresh™ (Intralytix)	*Salmonella* spp.	Food safety	Multi-phage cocktail for *Salmonella* reduction in foods	FDA GRAS (GRN 435)	[82]
Bafasal^®^ (Proteon Pharmaceuticals)	*Salmonella* spp.	Animal production	Veterinary phage feed additive for *Salmonella* reduction	European Union (2025/1390)	[83]
SalmoLyze™ (Intralytix)	*Salmonella* spp.	Pet food safety	Multi-lytic phage cocktail to reduce *Salmonella* contamination in pet food and raw feed materials	FDA (AGRN 74)	[84]
Salmopro™ (Phagelux)	*Salmonella* spp.	Poultry production	Two-strain lytic phage cocktail for on-farm and slaughterhouse poultry pathogen control	FDA GRAS (GRN 603)	[85]
Anti-*S. aureus* phage cocktail	*S. aureus*	Bovine mastitis treatment	Lytic phage cocktail for intramammary infusion	Field clinical trial completed (Germany)	[66]

**Table 4:** Challenges and future perspectives for phage therapy of bovine mastitis.

Challenge	Underlying Mechanism	Strategy	References
Host range limitation and phage resistance	RBP-mediated host recognition and the diversity of bovine mastitis pathogens; bacterial defense systems restrict phage efficacy	Phage cocktails; RBP engineering and depolymerases; phage–antibiotic synergy and co-evolution-guided phage selection	[51,58,64,68,98,99]
Mammary gland microenvironment	Milk fat globules, casein, immunoglobulins, proteases, pH changes reduce phage stability and activity	Encapsulation; lipid nanocarriers; polymeric microparticles; formulation optimization	[95,102,103,105]
Safety and ecological risks	Horizontal gene transfer (HGT); virulence factors; lysogeny; microbiota disruption and immune activation	Genomic screening; lytic phage selection; ISO-guided sequencing; long-term monitoring	[59,60,107,110]

## Data Availability

No new data were created or analyzed in this study. Data sharing is not applicable to this article.
